# Nervous Diseases

**Published:** 1875-05

**Authors:** E. H. Gibrs


### NERVOUS DISEASES.

BY E. H. GIBBS, A.M., M.D.

It is safe to affirm that most diseases may be classified as NERVOUS. The reason is obvious. The brain is the great nerve-center, and the spinal cord and thirty pairs of nerves that branch out from it, and the lesser branches of these thirty pairs, even to the smallest nerve-filament in the system, are portions of the brain, as the roots and branches are portions of the tree. Every emotion or sensation manifests itself through the medium of its appropriate nerves. They are the sleepless monitors that warn us of the approach of disease.

Hence when there is pain in any particular part of the body the attention is directed to the complaining nerve. But unless there is something more than the presence of pain at the point indicated, there is liability to errors of diagnosis. Pain is frequently transmitted from the actual seat of the disease to some point more or less distant, usually on the principal nerve near it. In cases of hip-disease, the severest and most persistent pain is usually in the knee. Ignorance of this fact would cause the treatment to be directed to the knee, while the disease in the hip-joint would be permitted to commit its ravages unheeded and unchecked. There is seldom a case of spinal irritation so well marked, but that the inexperienced will insist that it is dyspepsia or perhaps palpitation of the heart. These are merely the symptoms of the disease, readily recognized by the educated physician.

### SPINAL IRRITATION.

Some knowledge of the structure of the spine is necessary to a comprehension of its functions and diseases.

No part of the human anatomy should receive more careful study and attention; for it is the initial point of many of the most distressing and fatal diseases known to physicians. As before remarked, the spinal cord is connected with the brain. It passes down the entire length of the back, through its unyielding channel of bone, which it nearly fills. This channel is lined with a firm membrane called the "periostium." When irritated or inflamed, the periostium becomes swollen. This may diminish the calibre of the channel through which the spinal cord passes, sufficient to compress the cord and produce the symptoms. The irritation is usually confined to a few vertabræ. Sometimes there is great tenderness along the whole length of the spinal column. The symptoms vary with the portion of the spine affected; those organs especially showing signs of disorder, which receive nerves directly or indirectly from the vicinity of the tender vertebræ. There is usually derangement of the digestive functions, such as attend the worst cases of dyspepsia.

The respiratory organs are liable to be attacked. Palpitations of the heart are frequent, as well as other signs of functional cardiac disorder, such as faintness, and pains resembling *angina pectoris.* Other organs are variously affected. Neuralgic pains are felt in the muscles of the chest and shoulders. Pains resembling rheumatism, occur in various parts of the body.

In short, there is scarcely a morbid sensation or perversion of function, which may not originate in spinal irritation. In all cases where the cause of any existing disorder of this kind is obscure, it should be sought for in the spine. There is reason to believe that some cases resembling tetanus and hydrophobia, are in fact dependent on spinal irritation.

The disease occurs most frequently in women between the ages of 14 and 45, although neither sex is exempt. Whenever any of these symptoms make their appearance and persist in remaining for even a few days, the best medical advice should be sought. Prompt attention may save years of suffering. If neglected, spinal irritation may degenerate into

#### ORGANIC DISEASE OF THE SPINAL MARROW.

In this disease we find many of the symptoms of spinal irritation aggravated. Instead of a feeling of uneasiness along the spinal column, there will be pain more or less severe. Morbid sensations of a serious type will be found in those portions of the body having direct nerve connection with the diseased spine. Added to this, there is a gradually increasing weakness of the muscles, which at length ends in paralysis of sense and motion, and death. Happily the disease is rare. But the fact that it does occur, and with even more aggravated symptoms than those above described, should warn us to look well to the first indications of spinal trouble.

#### TREATMENT OF SPINAL IRRITATION.

There are few diseases that yield more readily to judicious treatment, than spinal irritation. It ordinarily requires only skill and experience in diagnosis and the application of the proper remedies. Of these the most important by far are counter irritants and the application of electricity. But it will not do to use these indiscriminately. Each case must be studied, and in each the appropriate remedies employed. Some cases require tonics, others do not. Some require regular and moderate exercise; to others exercise might be fatal. There is also great difference in the time required to restore the patient to health. Some are completely restored to health in 40 days; others require six months. We believe, however, that this depends much on the patient. One imprudent act may retard the treatment for months.

